# A functional SNP associated with atopic dermatitis controls cell type-specific methylation of the *VSTM1* gene locus

**DOI:** 10.1186/s13073-017-0404-6

**Published:** 2017-02-20

**Authors:** Dilip Kumar, Kia Joo Puan, Anand Kumar Andiappan, Bernett Lee, Geertje H. A. Westerlaken, Doreen Haase, Rossella Melchiotti, Zhuang Li, Nurhashikin Yusof, Josephine Lum, Geraldine Koh, Shihui Foo, Joe Yeong, Alexessander Couto Alves, Juha Pekkanen, Liang Dan Sun, Astrid Irwanto, Benjamin P. Fairfax, Vivek Naranbhai, John E. A. Common, Mark Tang, Chin Keh Chuang, Marjo-Riitta Jarvelin, Julian C. Knight, Xuejun Zhang, Fook Tim Chew, Shyam Prabhakar, Liu Jianjun, De Yun Wang, Francesca Zolezzi, Michael Poidinger, E. Birgitte Lane, Linde Meyaard, Olaf Rötzschke

**Affiliations:** 1 Singapore Immunology Network (SIgN), A*STAR (Agency for Science, Technology and Research), 8A Biomedical Grove #04-06, Singapore, 138648 Republic of Singapore; 2 Laboratory of Translational Immunology, Department of Immunology, University Medical Center Utrecht, P.O. box 85090, Utrecht, 3508 AB The Netherlands; 3 Department of Epidemiology and Biostatistics, School of Public Health, Imperial College London, London, UK; 4 Department of Environmental Health, National Institute for Health and Welfare, Kuopio, Finland; 5 Institute of Dermatology and Department of Dermatology at No.1 Hospital, Anhui Medical University, Hefei, Anhui China; 6 Genome Institute of Singapore (GIS), Agency for Science, Technology and Research of Singapore (A*STAR), Singapore, Republic of Singapore; 7 Wellcome Trust Centre for Human Genetics, Oxford, UK; 8 Department of Oncology, Cancer and Haematology Centre, Churchill Hospital, Oxford, UK; 9 Institute of Medical Biology (IMB), A*STAR (Agency for Science, Technology and Research), Singapore, Republic of Singapore; 10 Department of Otolaryngology, National University of Singapore, Singapore, Republic of Singapore; 11 Biological Sciences, National University of Singapore, Singapore, Republic of Singapore; 12 National Skin Center, Singapore, Republic of Singapore; 13 Center for Life Course Epidemiology, Faculty of Medicine, University of Oulu, P.O. Box 5000, 90014 Oulu, Finland; 14 Biocenter Oulu, University of Oulu, P.O. Box 5000, Aapistie 5A, 90014 Oulu, Finland; 15 Unit of Primary Care, Oulu University Hospital, Kajaanintie 50, 90029 OYS, P.O. Box 20, 90220 Oulu, Finland; 16 Department of Pathology, Singapore General Hospital, Singapore, Republic of Singapore; 17 Institute of Molecular & Cellular Biology (IMCB), Agency for Science, Technology and Research (A*STAR), Singapore, 138648 Republic of Singapore; 18 Department of Physiology, NUS Yong Loo Lin School of Medicine, National University of Singapore, Singapore, Republic of Singapore; 19 Department of Biological Sciences, National University of Singapore, Singapore, Republic of Singapore

**Keywords:** VSTM1, Signal inhibitory receptor on leukocytes-1 (SIRL-1), Expression quantitative trait loci (eQTL), Atopic dermatitis, Monocytes, Reactive oxygen species (ROS), Neutrophils

## Abstract

**Background:**

Expression quantitative trait loci (eQTL) databases represent a valuable resource to link disease-associated SNPs to specific candidate genes whose gene expression is significantly modulated by the SNP under investigation. We previously identified signal inhibitory receptor on leukocytes-1 (SIRL-1) as a powerful regulator of human innate immune cell function. While it is constitutively high expressed on neutrophils, on monocytes the SIRL-1 surface expression varies strongly between individuals. The underlying mechanism of regulation, its genetic control as well as potential clinical implications had not been explored yet.

**Methods:**

Whole blood eQTL data of a Chinese cohort was used to identify SNPs regulating the expression of VSTM1, the gene encoding SIRL-1. The genotype effect was validated by flow cytometry (cell surface expression), correlated with electrophoretic mobility shift assay (EMSA), chromatin immunoprecipitation (ChIP) and bisulfite sequencing (C-methylation) and its functional impact studied the inhibition of reactive oxygen species (ROS).

**Results:**

We found a significant association of a single CpG-SNP, rs612529T/C, located in the promoter of *VSTM1*. Through flow cytometry analysis we confirmed that primarily in the monocytes the protein level of SIRL-1 is strongly associated with genotype of this SNP. In monocytes, the T allele of this SNP facilitates binding of the transcription factors YY1 and PU.1, of which the latter has been recently shown to act as docking site for modifiers of DNA methylation. In line with this notion rs612529T associates with a complete demethylation of the *VSTM1* promoter correlating with the allele-specific upregulation of SIRL-1 expression. In monocytes, this upregulation strongly impacts the IgA-induced production of ROS by these cells. Through targeted association analysis we found a significant Meta *P* value of 1.14 × 10^–6^ for rs612529 for association to atopic dermatitis (AD).

**Conclusion:**

Low expression of SIRL-1 on monocytes is associated with an increased risk for the manifestation of an inflammatory skin disease. It thus underlines the role of both the cell subset and this inhibitory immune receptor in maintaining immune homeostasis in the skin. Notably, the genetic regulation is achieved by a single CpG-SNP, which controls the overall methylation state of the promoter gene segment.

**Electronic supplementary material:**

The online version of this article (doi:10.1186/s13073-017-0404-6) contains supplementary material, which is available to authorized users.

## Background

Genome-wide association studies (GWAS) in the recent past have identified thousands of disease-related genetic variants and trait-associated polymorphisms [1–3]. The National Genome Research Institute (NHGRI) GWAS catalog alone comprises data of more than 2000 different studies organized with association *P* values from hundreds of disease phenotypes and complex traits [4]. However, translating these associated variants to causality and potential mechanisms of disease have been difficult. Recent advances in high throughput genotyping and gene expression analysis have allowed for an extensive mapping of expressed quantitative trait loci (eQTL) [5, 6]. Although many of these eQTL are cell-type–specific and also vary between the ethnic groups, they represent a valuable resource for candidate proteins, whose messenger RNA (mRNA) expression is, at least in part, genetically controlled [5–7]. These eQTL datasets provide an excellent opportunity to address and understand the mechanisms underlying GWAS hits. Zhu et al. have recently demonstrated this at a whole genome level by providing a list of priority candidate genes for further functional evaluation of the five complex human traits [8]. However, once the priority candidate genes have been identified, it needs to be validated using well designed in vitro, in vivo, or ex vivo models using techniques, which go beyond assaying the genome and transcriptome [9, 10]. Additionally, this also requires knowledge on the protein molecule under investigation or pathways identified, cell/tissue specificity, and functional consequence on the immune system. Hence, there is a need for targeted but yet comprehensive candidate gene-based studies looking at various aspects of the potential disease gene from DNA to protein, also in pure populations of immune cells and/or tissues with appropriate stimuli and functional readouts. In this study, we present an in-depth, well-characterized study of the *SIRL-1* (signal inhibitory receptor on leukocytes-1) gene using various complementary techniques.

The “immune inhibitory receptors” represent a heterogeneous family of transmembrane receptors that function as negative regulators of immune cells [11, 12]. Upon ligation, the inhibitory signal is transduced through immunoreceptor tyrosine-based inhibitory motifs (ITIMs) located in the cytoplasmic tail of the receptor [13]. One member of this family is SIRL-1. While the natural ligand is not known yet, cross-linking of the receptor with agonistic antibodies results in the tyrosine phosphorylation of the ITIMs by the Src family kinases (SFK). The phosphorylated ITIMs act as a docking site for the recruitment of the Src homology 2 (SH2) domain-containing tyrosine phosphatases SHP-1 and/or SHP-2[14]. These phosphatases antagonize the effects of activating SFK and Syk family kinases, triggered for instance by the engagement of IgE-specific Fc-epsilon receptors [15]. Therefore, these immune inhibitory receptors have been implicated to play a role in regulating antibody-mediated immune responses. SIRL-1 is known to primarily express on human myeloid cells, where it inhibits crucial pro-inflammatory functions, such as Fc receptor (FcR) mediated respiratory burst and neutrophil extracellular trap (NET) formation [12, 14, 16, 17]. In a prior study, we made an observation reporting striking differences in the expression level of SIRL-1 on various immune subsets. Granulocyte subsets neutrophils and eosinophils constitutively expressed high levels SIRL-1 on most individuals, while the expression level of the receptor on monocytes varied strongly between individuals [16]. The cause of these variations as well as the potential clinical implications was not known.

In this study, we used mRNA and genotype data from two independent cohorts (Chinese, Caucasian) to link population variations of the molecule on monocytes to a single single nucleotide polymorphism (SNP), rs612529T/C, a CpG-SNP located in the promoter region of the SIRL-1 encoding gene *VSTM1*. By employing flow cytometry (FACS), functional assays, electrophoretic mobility shift assay (EMSA), bisulfite DNA sequencing, and chromatin immune precipitation (ChIP) analysis, we could further show that SIRL-1 upregulation correlates with the allele dependent binding of PU.1 and YY1 to the CpG-SNP, which in turn is associated with a demethylation of the entire promoter region. Importantly, genetic association analysis suggested that the cell-type–specific fluctuations of SIRL-1 indeed might have pathologic consequences. We observed that in three independent cohorts, the C allele (indicative of low expression) is consistently associated with an increased risk for atopic dermatitis (AD), an inflammatory skin condition.

## Methods

### Human blood samples and cohorts

The cohorts used in this study were outlined in Additional file 1. eQTL analysis was carried out with ethnic Chinese cohorts collected in Singapore (“blood eQTL cohort” and “monocyte & B cell eQTL cohort”) of 202 (unpublished) and 15 individuals [18], respectively. In addition, data were used from a “Monocyte & B cell cohort” of 279 individuals of Caucasian ethnicity that had been reported before by Fairfax et al. [19] With the exception of the “functional cohort” (see below), details on all cohorts had been published previously. Data of three additional cohorts (Singapore Chinese population [20], Chinese Han Population [21] and Caucasian population [22]) that had been published previously were used for the genetic association of the VSTM1-SNP rs612529 with AD.

### Functional cohort

Another genotyped Singaporean cohort consisting of ethnic Chinese individuals was used for the functional characterization of VSTM1 by FACS (n = 44), reactive oxygen species (ROS) assay (n = 30), DNA-methylation (n = 8), and ChIP assay (n = 9) (“functional cohort”). For all individuals of the “functional cohort,” the rs612529T/C genotype was determined by using high resolution melting (HRM) analysis. The functional cohort was used for the rs612529 genotype/phenotype association, measurement of NADPH oxidase mediated ROS production assay, ChIP assay and bisulfite treatment, and methylation analysis as described below.

### Genotyping

DNA was isolated from whole blood or peripheral blood mononuclear cells (PBMCs) using DNeasy Blood & Tissue Kits (Qiagen) according to the manufacturer’s instructions. For targeted genotyping, the HRM analysis was performed by real-time polymerase chain reaction (PCR) using a CFX96 Real-Time Detection System (Bio-Rad). The primers used for rs612529 genotyping were 5′-TCTTGGCAGAACTTCAGATAAGGT-3′ (forward) and 5′-ACAAGAAGCCGTCGATGATAACT-3′ (reverse). Amplification was carried out using the following protocol: 3 min at 95 °C, 40 cycles of 5 s at 95 °C, 5 s at 50 °C, and final extension for 10 s at 95 °C. A melting curve was generated in the range of 65–95 °C (in 0.2 °C increments) with 10 s/step. Heterozygosity and homozygosity of the allelic state were deduced using Precision Melting Analysis software (Bio-Rad).

### RNA extraction and global gene expression for whole blood samples

Total RNA was extracted from human whole blood collected in Tempus Blood RNA tubes (ThermoFisher) using the MagMAX™ for Stabilized Blood Tubes RNA Isolation Kit (ThermoFisher). Total RNA was subjected to globin mRNA depletion using the Ambion GLOBINclear kit (ThermoFisher). Agilent Bioanalyzer was used to evaluate total RNA integrity and the RNA Integrity Number (RIN) was calculated. All samples had RIN in the range of 4.7–8.8; however, the median value was 7. For genome-wide gene expression profiling, fluorescent-labeled PCR products were prepared according to Illumina Human Whole-Genome Gene Expression DASL Assay Guide. The labeled products were hybridized onto the Illumina HumanHT-12-v4 Expression Bead ChIP for 16 h at 58 °C. These arrays were washed, coated based on the Assay Guide, and scanned using Bead Array Scanner 500GX at BSF Microarray Facility.

### eQTL analysis

The whole blood cohort used for eQTL analysis has not been published yet. The median age is 21 years and 55% male. Whole blood gene expression data were processed using the Bioconductor lumi package in R 3.1.2. The gene expression data were quantile normalized and log2 transformed prior to further analysis. Whole blood gene expression data for genes located 200kbp upstream and downstream of the SNP rs612529 were extracted and combined with the SNPs located within this same region. One-way ANOVAs were then done to determine if any of the SNP genotypes associated with the gene expression levels. The one-way ANOVA was conducted in R 3.1.2 and all resulting *P* values were corrected for multiple testing using the method of Benjamini and Hochberg. The eQTL data for the B cells and monocytes for the 15 samples for the Chinese samples from Singapore were previously described in Melchiotti et al. [18]. Data for the B cells and monocytes for the Caucasian cohort were extracted from Fairfax et al. [19].

### Association analysis for rs612529

The association analysis for the SNP rs612529 was performed using statistics from the various cohorts using Stouffer z-trend method using the Meta *P* program (http://igm.cumc.columbia.edu/MetaP/metap.php). The Stouffer’s z trend method considers *P* values, sample sizes, and effect directions. For more information on the program, refer to Whitlock et al. [23].

### Flow cytometry analysis

Blood samples were collected in either BD K_2_EDTA or citrate vacutainer tubes. FACS staining was performed on either using whole blood sample or isolated PBMCs. Erythrocytes were removed from whole blood by incubation in a 14-mL erythrocyte lysis buffer (155 mM NH_4_Cl, 10 mM KHCO_3_, 0.1 mM EDTA) for 10 min at room temperature. After erythrocytes lysis, the cells were centrifuged at 860 × g for 3 min. The cell pellets were re-suspended in phosphate-buffered saline (PBS) and washed once with PBS by centrifugation at 860 × g for 3 min at room temperature. For PBMC isolation, whole blood was layered on Ficoll-Paque (GE Healthcare) and centrifuged for 500 × g for 30 min without a break. PBMCs were harvested at the interface between Ficoll and plasma layers. To discriminate the cells live from the dead cells, lyzed whole blood samples or PBMC cell pellets were incubated with 100 μL of LIVE/DEAD Fixable Aqua Dead kit (Life Technologies) in PBS for 10 min at room temperature. The cells were washed once with 100 μL MACS buffer (0.5% BSA, 2 mM EDTA in PBS) and transferred into 96 V-bottom plates for centrifugation at 1000 × g for 3 min. For the SIRL-1 phenotyping, the cell pellets were incubated with for 15 min at 4 °C anti-SIRL-1 FITC (1A5; as previously described [14]), anti-FcεRI PE (AER37; eBioscience), anti-CD123 PerCP Cy5.5 (6H6; eBioscience), anti-CD1c BV421 (L161; Biolegend), anti-CD14 PE-CF594 (MΦP9; BD Biosciences), anti-CCR3 Alexa Fluor647 (5E8; Biolegend) and anti-HLA-DR APC-H7 (L243; BD Biosciences). For the correlation of SIRL-1 with Fc receptor expression, the staining was carried out with anti-FcεR1 (AER37), anti-CD16 (3G8; Biolegend), and anti-CD89 (A59; BD Bioscience) monoclonal antibodies. After incubation, the cell pellets were washed once with MACS buffer, re-suspended in the 250 μL of the same buffer, and SIRL-1 expression level on different cell types was determined using a LSRII or a Fortessa flow cytometer (BD Biosciences).

### Cell isolation

Monocytes used for nuclear protein extraction were isolated from apheresis blood and for ChIP and bisulfite sequencing monocytes were isolated from PBMCs derived from functional cohort by positive selection using MACS Human CD14 Microbeads (Miltenyi Biotec) according to the instructions recommended by manufacturer. Neutrophils used for the bisulfite sequencing were isolated by Ficoll-Paque density gradient centrifugation. The neutrophils were isolated from the layer immediately above erythrocyte layer and lyzing the erythrocyte with erythrocyte lysis buffer (155 mM NH_4_Cl, 10 mM KHCO_3_, 0.1 mM EDTA) for 10 min at room temperature on a roller mixer. The neutrophils were washed once with cold RMPI1640 supplemented with 10% fetal bovine serum.

### Measurement of NADPH oxidase mediated ROS production

ROS production was measured in primary monocytes isolated from frozen PBMC samples of genotyped donors of the functional cohort. All experiments were performed using technical duplicates. NADPH oxidase activity was, unless otherwise indicated, assessed as H_2_O_2_ formation and determined by Amplex Red (Molecular Probes), which reacts with H_2_O_2_ to produce fluorescent resorufin in the presence of HRP (Sigma-Aldrich). The assay was performed in microfluor white plates (Thermo Scientific). Isolated monocytes were re-suspended in HEPES buffer (20 mM HEPES, 132 mM NaCl, 6 mM KCl, 1 mM MgSO_4_, 1.2 mM KH_2_PO_4_, pH 7.4 supplemented with 0.5% human serum albumin (Sigma), 1 mM Ca2+, and 5 mM D-glucose) containing 50 μM Amplex Red and 1 U/mL HRP and stimulated (1 × 10^6^ cells/mL) with 10 μg/mL plate-bound human IgA (ChromPure Human IgA, Jackson ImmunoResearch Laboratories) together with 10 μg/mL plate-bound anti-SIRL-1 (1A5) or an isotype-matched control mAb (functional grade mouse IgG clone:P3, eBioscience). Formation of H_2_O_2_ was measured directly from the start of stimulation, when cells were added to coated wells, and continued for 1 h. Fluorescence was measured at 1-min intervals (λ_Ex_/λ_Em_ = 545/590 nm) using a Tecan Infinite M200 PRO fluorescence reader (Tecan). Measurements were corrected for spontaneous H_2_O_2_ production, as determined by incubation of cells in non-coated wells. The percentage of SIRL-1-mediated inhibition of ROS production was calculated by sub-traction of background H_2_O_2_ production and subsequent calculation of the area under the curve of all samples. The AUC of IgA co-coated with an isotype-matched control mAb was set at 100% and compared with the AUC of IgA co-coated with anti-SIRL-1 mAb.

### Nuclear protein isolation

Freshly isolated monocytes from leukapheresis blood were washed with ice cold PBS, then buffer A (20 mM HEPES, pH 7.9, 20% Glycerol, 10 mM NaCl, 0.2 mM EDTA (pH 8), 1 mM DTT, 0.1% Triton X-100) supplemented with Pierce Proteases and Phosphatase Inhibitor Mini Tablets. The tablet contained aprotinin, bestatin, E-64, EDTA, leupeptin, sodium fluoride, sodium orthovanadate, sodium pyrophosphate, beta-glycerophosphate, and metalloproteinase inhibitor. After 15 min incubation on ice, homogenates were centrifuged at 2000 rpm at 4 °C for 15 min, and the resultant “nuclear pellets” were re-suspended in buffer A containing 500 mM NaCl. The nuclear proteins were incubated for 1 h on ice with intermittent tapping followed by centrifugation at 13,000 rpm (4 °C) for 15 min. The supernatants were then aliquoted and snap-frozen at –80 °C until used. Quantitation of nuclear proteins was performed using Bio-Rad protein quantitation kit (Bio-Rad).

### Electrophoretic mobility shift assay

The double-strand oligonucleotides had a length of 25 bp (the sequences are listed in Additional file 2). Allele-specific oligonucleotides corresponding to VSTM-1 promoter region –34 to –58 were designed with the C/T polymorphism of rs612529 in the center (termed as “C probe” and “T probe”). C and T probes, as well as MZF-1 consensus/mutant oligonucleotides, were synthesized by 1st BASE, Singapore; all remaining oligonucleotides were purchased from Santa Cruz Biotechnology. ^32^P labeling was introduced using T4 Polynucleotide Kinase. For EMSA reactions, 5 μg of the monocyte nuclear protein extract was incubated with 40,000 cpm of the ^32^P-labeled oligonucleotide probe in a total volume of 20 μL 5 mM HEPES (pH 7.9), 5% glycerol, 0.5 mM EDTA, 1 μg dIdC, 1 mM DTT, and 80 mM NaCl at 4 °C for 45 min. For “cold” competition experiments, 12.5–50 ng of the unlabeled oligonucleotide competitor 15 min were added before adding the respective radiolabeled probe, representing a 12.5–50-fold excess of the unlabeled competitor. For supershift assays, 2 uL of either rabbit polyclonal anti-PU.1 IgG (H-135 X, Santa Cruz Biotechnology) or rabbit polyclonal anti-YY1 IgG (C-20, Santa Cruz Biotechnology) or rabbit IgG (Abcam) were added 15 min before adding the respective radiolabeled probe. After incubation, the DNA–protein complexes were separated for 90 min with a 5% non-denaturing polyacrylamide gel at 240 V on a protein II gel-apparatus (Bio-Rad) using 0.5X Tris/Borate/EDTA (TBE). After electrophoresis, the gel was transferred to a Whatman paper and dried on a vacuum gel dryer (Gel Dryer 583, Bio-Rad) for 2 h at 80 °C. The dried gel was then exposed on an imaging plate and scanned using a FLA-5000 phospho-image scanner (Fujifilm Life Science).

### ChIP assay

ChIP assays were performed on monocytes isolated from four CC-donors and five TT-donors (rs612529) of the functional cohort. ChIP was carried out essentially as previously described [24]. Briefly, DNA/protein cross-linking was achieved by incubating the cells for 8 min at 37 °C in 1% formaldehyde. Ultrasound sonication of the chromatin was performed in lysis buffer (50 mM Tris-Cl pH 8.0, 10 mM EDTA, 1% SDS) with 3-min cycles (30 s “ON”, 30 s “OFF”) for a total of ten cycles using a Bioruptor UCD-300 sonicator (Diagenode). After sonication, samples were diluted in fivefold dilution buffer (0.01% SDS,1.1% Triton X-100, 1.1 mM EDTA, 20 mM Tris-Cl pH 8.0) followed by overnight incubation at 4 °C with 10 μg of either rabbit polyclonal anti-PU.1 antibody IgG (H-135 X, Santa Cruz Biotechnology), or rabbit polyclonal IgG (Abcam). Real-time PCRs of genomic regions containing the putative PU.1-binding site were performed in triplicate by using iTaq SYBR green supermix (Bio-Rad) with SIRL-1 specific primers (forward primer: 5′-TCTTGGCAGAACTTCAGATAAGGT-3′; reverse primer: 5′-ACAAGAAGCCGTCGATGATAACT-3′). The relative occupancy of the immunoprecipitated factor at SIRL-1 locus is estimated by using the comparative threshold method 2ˆ(Ct_mock_–Ct_specific_), where Ct_mock_ and Ct_specific_ are mean threshold cycles of PCR done in triplicate on DNA samples from mock and specific immunoprecipitation [24].

### Bisulfite treatment and DNA methylation analysis

Primers for the bisulfite genomic sequencing (BGS) were designed to cover a region 361 bp upstream and 335 bp downstream to the transcription start site of VSTM1 (Additional file 3). DNA bisulfite treatment of the genomic DNA isolated from freshly isolated monocytes and neutrophils of respectively four donors of the CC and TT genotype (functional cohort) was performed according to the protocol described in EZ DNA Methylation-Direct™ Kit (Zymo Research). For BGS, bisulfite-treated DNA was amplified using the SIRL-1 promoter specific primers

(F1: 5′-AGGTTGGAGTGTAGTGGTATAATTTTGG-3′,;

F2: 5′- ACTCTAACACTCTAATTCTCTACCCCAC-3′,;

B1:5′ AGAGTGGGGTAGAGAATTAGAGTGTTA and

B2: 5′- ACCTAAACTACCACTCCCACATAAAT-3′) with optimized conditions. The PCR products were cloned into the TA cloning vector (Invitrogen, Life technologies). For both monocytes and neutrophils three clones from each donor and sequenced using M13 forward primer.

### CpG islands (CGI) prediction

A Hidden Markov Model (HMM) based CGI prediction for the VSTM1 gene locus was obtained from UCSC genome browser as custom tracks (http://genome.ucsc.edu/cgi-bin/hgTracks) [25]. CpG islands (CGI) from DNA sequences and custom tracks were uploaded from Rafael Irizarry lab (http://rafalab.jhsph.edu/CGI/).

## Results

### *VSTM1* promoter-associated SNP rs612529 is an eQTL for SIRL-1

**Fig. 1:**
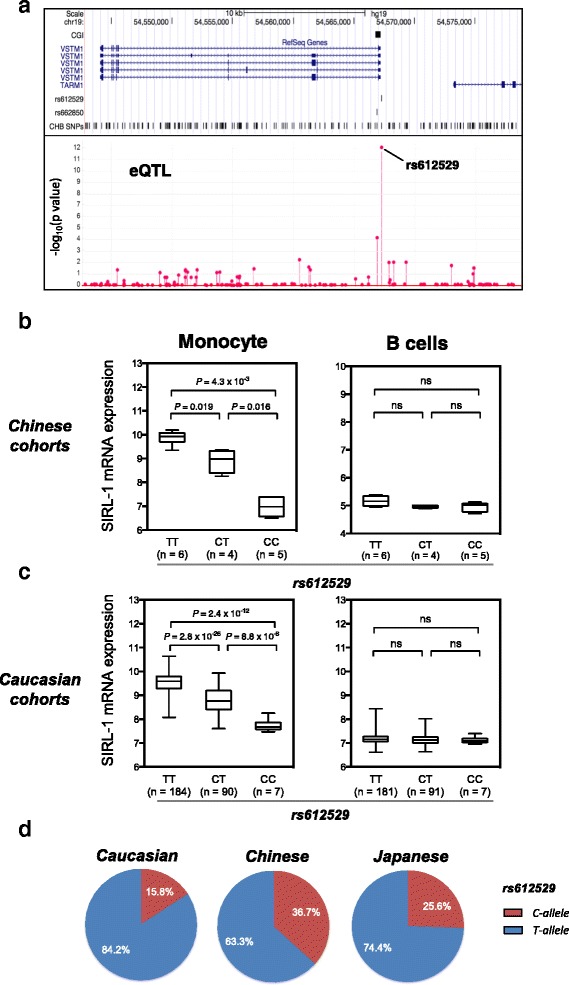
rs612529 is an eQTL affecting SIRL-1 expression. **a** *Manhattan plot* of whole blood eQTLs located in the SIRL-1-encoding *VSTM1* gene region. The *upper panel* depicts the position of *VSTM1* transcripts in relation to a CGI and the SNPs rs612529 and rs662850. The *lower panel* shows the eQTL-association determined from whole blood samples of 202 Chinese individuals. The *P* value (expressed as –log_10_) is displayed on the *y-axis*, the *x-axis* shows the location of the SNPs (rs612529 is indicated). **b**, **c** Genotype effect of rs612529 on the SIRL-1 mRNA expression monocytes and B cells. The data were generated from cohort samples of 15 Chinese [18] and 281 Caucasian individuals [19]. The number of individuals per genotype is indicated on the *x-axis*; the *y-axis* represents the SIRL-1 mRNA expression levels depicted as log_2_ value. **d** Allele frequencies of rs612529 in three major ethnical groups. The *pie charts* display the frequencies of the C allele (*red*) and the T allele (*blue*) for Caucasian, Han Chinese, and Japanese based on HapMap

We used whole blood eQTL data of 202 ethnic Chinese donors (unpublished dataset) collected in Singapore to determine if our previously reported inter-individual variations in SIRL-1 levels [12, 16] are linked to SNPs. The results described in Fig. 1a reveal a strong eQTL peak at the promoter of the *VSTM1* gene that encodes for SIRL-1 (all cohorts used in the study are described in Additional file 1). The effect was caused by a single SNP (rs612529T/C) located 45 bp upstream of the transcriptional start site (TSS) in the promoter region of *VSTM1* (*P* = 8.13 × 10^–13^). We also used our previous data [18] based on gene array analysis of monocytes and B cells isolated from a genotype matched Chinese cohort of 15 individuals. Primary isolated monocytes from the T allele donors exhibited a higher level of SIRL-1 mRNA expression, while the level was considerably lower in the C allele donors (Fig. 1b). The mRNA levels gradually increased from the CC over CT to TT genotype, resulting in a more than tenfold difference between the two homozygous genotypes of CC versus TT (*P* = 4.30 × 10^–3^). We validated this finding in a much larger dataset of 283 Caucasian individuals based on published eQTLs [19] (*P* = 2.40 × 10^–12^) (Fig. 1c). The low expression allele was less frequent in the Caucasians with allele frequency of the C allele in Caucasian at 15.8% compared with 36.7% in Chinese and 25.6% Japanese (Fig. 1d). No significant eQTL association was observed for *VSTM1* mRNA in B cells isolated from the same donors in both datasets (Fig. 1b and c).

### Cell-type–specific regulation of SIRL-1 protein expression in monocytes

**Fig. 2:**
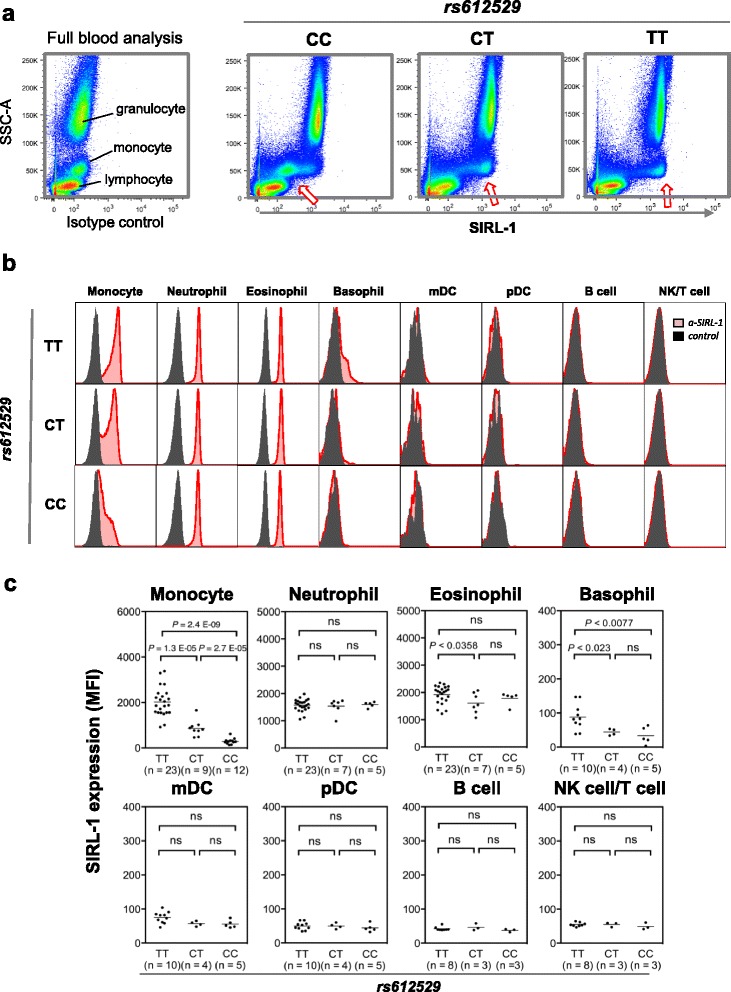
Cell-type–specific effect of rs612529 on SIRL-1 surface expression. **a** *Flow cytometry analysis* of whole blood samples. Whole blood samples were stained with a SIRL-1 specific antibody (*right panel*) and an isotype-matched control antibody (*left panel*). The staining is shown vs. the side scatter (SSC-A), which allows a simple discrimination of granulocytes, monocytes, and lymphocytes. The *plots* are representative examples of individuals with the rs612529 genotype TT, TC, and CC. *Arrows* indicate the gradual increase in SIRL-1 staining on monocytes. **b** Genotype-dependent SIRL-1 expression on various cell types. SIRL-1 staining (*red*) in reference to the isotype control (*black*) is shown for each of the three rs612529-genotypes for myeloid cells (monocyte, neutrophil, eosinophil, basophil, mDC, and pDC) and two lymphocyte subsets (B cell, NK cell/T cell). Data were generated by flow cytometry from whole blood samples after gating on the respective cell subset (gating strategy is displayed in Additional file 4). **c** Cohort-wide distribution of the SIRL-1 expression. The *dot plots* summarize the SIRL-1 FACS data for a cohort of 44 genotype-matched individuals

Our eQTL association for rs612529 seems to affect *VSTM1* expression monocytes and not B cells. SIRL-1 is expressed on various immune cells of the myeloid lineage. To determine the effect of rs612529 monocyte SIRL-1 at the protein level, we used flow cytometry to analyze three genotype donors (Fig. 2a). Scatter analysis with anti-SIRL-1 staining revealed allele-dependent effects on the amount of SIRL-1 surface expression only on monocytes. Notably, no influence of the SNP was observed for the granulocyte subset (Fig. 2a). On these cells a high expression of the receptor was detected independent of the genotype of rs612529. We then validated the finding in a cohort of 44 donors by using a staining panel of seven antibodies (Fig. 2b). The gating strategy (Additional file 4) allowed the simultaneous quantification of the SIRL-1 expression on various cells of the myeloid lineage (monocytes, neutrophils, eosinophils, basophils, myeloid dendritic cells (mDC), and plasmacytoid DC (pDC) as well as on lymphocytes (B cells, natural killer (NK) cells/T cells) (Fig. 2c). The analysis confirmed the strong genetic association with SIRL-1 surface expression on monocytes (*P* = 2.4 × 10^–9^) as well as the high but genotype-independent expression on neutrophils. A slight effect of the rs612529 polymorphism was observed also on eosinophils and basophils (*P* = 0.0358 and *P* = 0.0077, respectively), while on mDC, pDC, B cells, and NK cells/T cells, the SIRL-1 expression fell below detection limit (Fig. 2c).

### Impact of rs612529 on the inhibition of ROS

**Fig. 3:**
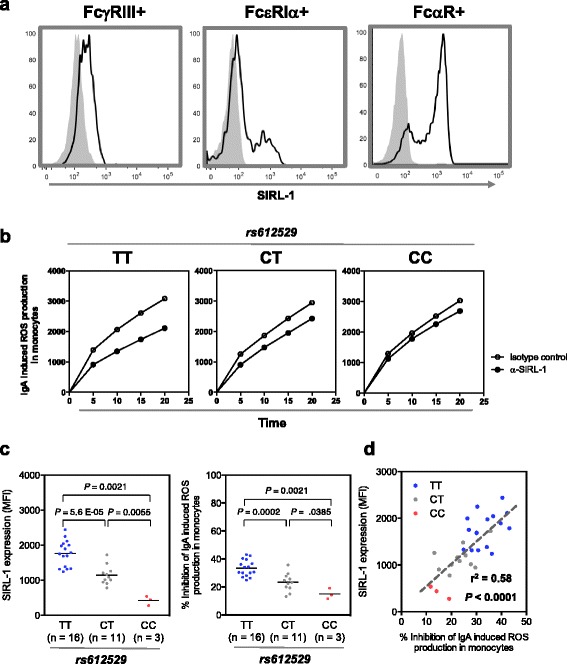
Allele-specific inhibition of IgA-induced ROS production by SIRL-1. **a** Comparison of the SIRL-1 expression on monocytes expressing FcγRIII-, FcεRIa- or FcαR-receptors. *Flow cytometry analysis* of the SIRL-1 surface expression is shown for monocytes gated on the expressing of the Fc-receptors FcγRIII, FcεRI or FcαR (gating strategy is shown in Additional file 5). The SIRL-1 specific staining (*black lines*) is shown in reference to the isotype control (*gray*). **b** Allele-dependent inhibition of ROS production. FcαR-mediated ROS production is shown for primary monocytes induced by plate-bound IgA in the presence of agonistic anti-SIRL-1 antibodies (*filled circle*) or isotype-matched control antibody (*open circle*). Representative examples are shown for each of the three rs612529-genotypes. ROS production was measured by ELISA at λ_Ex_/λ_Em_ = 545/590 nm using H_2_O_2_ sensitive Amplex Red; the units represent background corrected relative fluorescence unit (RFU). **c** ROS inhibition obtained for a cohort of 30 genotyped donors. The plots display the SIRL-1 surface expression (*left panel*) and the percent inhibition of the IgA-induced ROS production (*right panel*) in reference to the respective rs612529 genotype. **d** The correlation of SIRL-1 expression with the inhibition of IgA-induced ROS production. The correlation shown is 30 individuals. *Dashed line* represents the linear regression. rs612529 genotype is indicated by the color code (TT: *blue*, CT: *gray*; CC: *red*)

We have shown previously that SIRL-1 inhibits the FcR-mediated respiratory burst [12, 16]. In order to determine the functional implications of the polymorphism, we analyzed the antibody-induced ROS production in monocytes based on the genotype of rs612529. Depending on differentiation and maturation, monocytes express varying amounts of FcR specific for IgG (FcγRIII, CD16), IgE (FcεRI), and IgA (FcαR, CD89). Our flow cytometry analysis on ex vivo monocytes revealed that the SIRL-1 expression was highest on monocytes expressing the Fc receptor for IgA (Fig. 3a, Additional file 5). More than 90% of the FcαR+ cells co-expressed SIRL-1 (Fig. 3a, right panel). Hence, for the follow-up ROS analysis, the FcR-induced respiratory burst was induced using immobilized IgA. The resulting data, as shown in Fig. 3b, demonstrate that exposure of primary monocytes to IgA resulted in a strong production of ROS. Simultaneous ligation of SIRL-1 with an agonistic antibody resulted in an rs612529 allele-dependent reduction of the levels of detectable ROS. This was confirmed with monocytes isolated from a cohort of 30 genotyped individuals (Fig. 3c). A gradual decrease in the inhibition from TT over TC to CC was observed, a trend that matched the decreasing amount of SIRL-1 on the surface of these cells. This was particularly evident in a direct correlation between the amount of SIRL-1 on the surface of the cells and the inhibition of the IgA-induced ROS production (Fig. 3d).

### rs612529 regulates the recruitment of PU.1 and YY1 to the *VSTM1* promoter

**Fig. 4:**
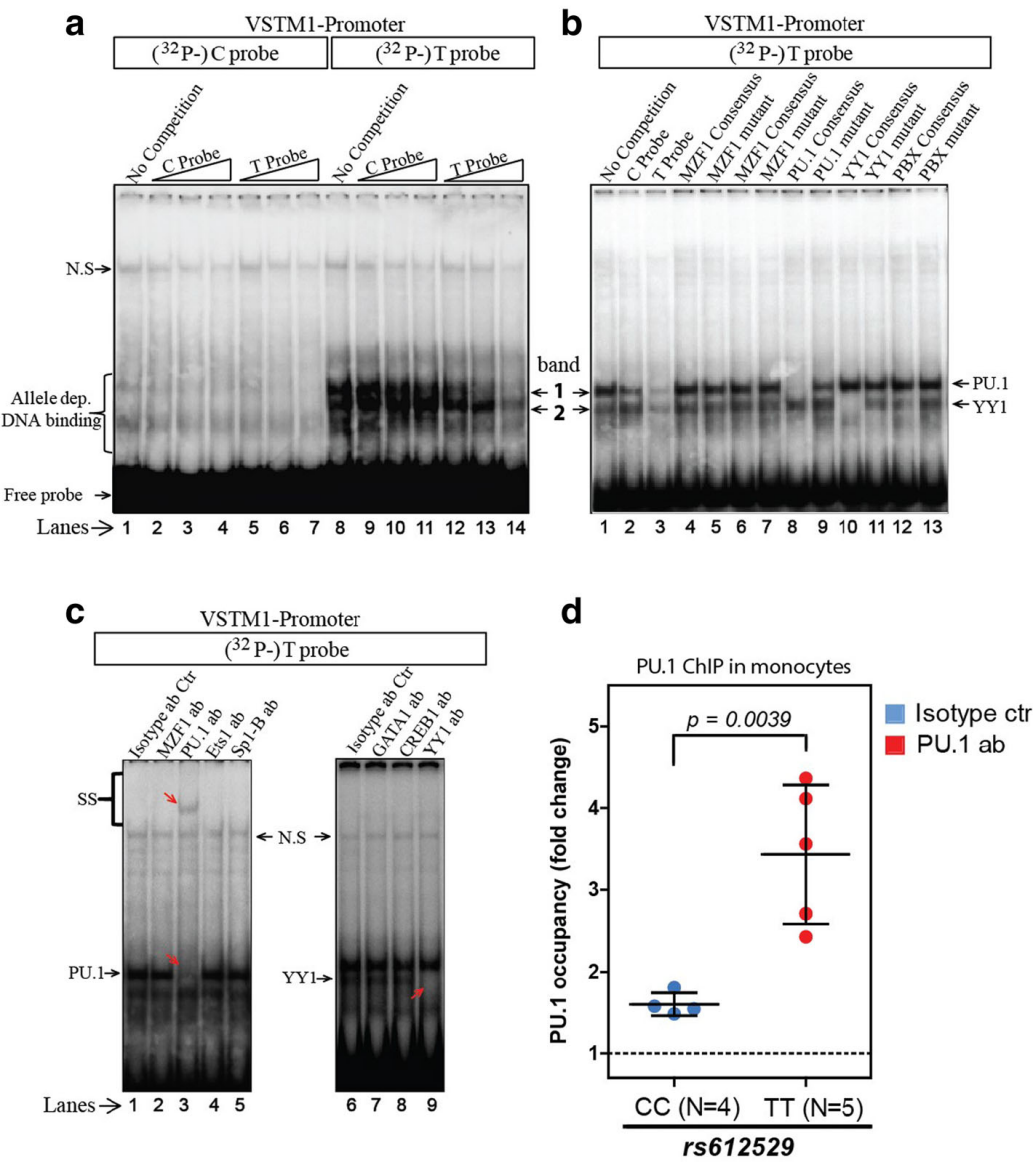
rs612529 C/T controls the binding of PU.1/YY1 to the VSTM-1 promoter. **a** Allele-specific binding to rs612529. EMSA experiments with nuclear extracts from monocytes were carried out with radiolabeled probes representing the C allele (“C probe;” lanes 1–7) or the T allele of rs612529 (“T probe;” lanes 8–14). In a “cold” competition, the binding to the radiolabeled probes was competed with unlabeled C probes (lanes 2–4 and 9–11) or T probes (lanes 5–7 and 12–14). Bands 1 and 2 are two bands formed by allele-specific binding; “N.S.” indicates a non-specific band. **b** Competition with consensus binding motifs of predicted candidate factors. Binding to the T probe was competed with unlabeled probes representing consensus binding motifs for MZF-1, PU.1 and YY1 (PBX was used as control). Binding of each factor was competed with the optimal consensus sequence as well as a mutated variant to which the binding was abolished (sequences are listed in Additional file 7). **c** Super-shift assays. Super-shift assays were carried out with the T probe and antibodies against MZF-1 (lane 2), PU.1 (lane 3), Ets1 (lane 4), Sp1-B (lane 5), GATA1 (lane 7) CREB1 (lane 8), and YY1 (lane 9) or isotype-matched control antibodies (lanes 1 and 6). Super-shifted bands and bands lost by the antibody treatment are indicated by *red arrows*. **d** PU.1-ChIP assay. A ChIP assay with nuclear samples from monocytes of four CC and five TT donors (rs612529) was carried out with PU.1-specific antibodies (*red dots*) or isotype-matched control antibodies (*blue dots*). PU.1 occupancy, depicted as fold change compared to the isotype, was determined by quantitative PCR amplification of the respective region in the VSTM-1 promoter (Additional file 3)

In order to determine if rs612529 modulates the binding of essential transcription factor(s) we carried out EMSAs. EMSA experiments performed with monocyte-derived nuclear samples revealed allele-specific binding of at least two different factors (Fig. 4a). Both factors bound selectively to the T allele (“T probe”) but not the C allele (“C probe”) of an oligonucleotide probe representing the 21 bp promoter segments of the SNP. Allele specificity of the binding was confirmed by cold competition experiments, since unlabeled T probe could out-compete both factors, while the unlabeled C probe had no effect on their binding to the ^32^P-labeled T probe.

In silico analysis by JASPAR (http://jaspar.binf.ku.dk/) and TRANSFAC (http://www.gene-regulation.com/) predicted binding by PU.1, MZF-1, and YY1 (Additional file 6). EMSA competition of the radiolabeled T probe with unlabeled oligonucleotides representing the consensus binding sequences of these transcription factors suggested band 1 to be formed by PU.1, while band 2 derived from YY1 (Fig. 4b; Additional files 2 and 7). The identity of the two factors was formally confirmed by super-shift assays, in which the PU.1-specific and YY1-specific antibodies super-shifted and/or blocked the complex formation with the T probe (Fig. 4c).

The allele-specific interaction of PU.1 with the *VSTM1* promoter region in monocyte-derived nuclear samples was also confirmed in ChIP assays (Fig. 4d). DNA fragments precipitated from four CC and five TT donors with PU.1-specific antibodies were analyzed by quantitative PCR with a primer pair covering a region of the *VSTM1* promoter including the rs612529 SNP (Additional file 3). In line with the results from the EMSA experiments, a significant increase in the amount of *VSTM1* DNA was detected in the PU.1 precipitate from TT donors compared to CC donors (Fig. 4d). Thus, in monocytes PU.1 is strongly associated with the analyzed *VSTM1* promoter segment when rs612529 consists of the T allele.

### The allelic state of rs612529 is correlated with the methylation of *VSTM1* in monocytes

**Fig. 5:**
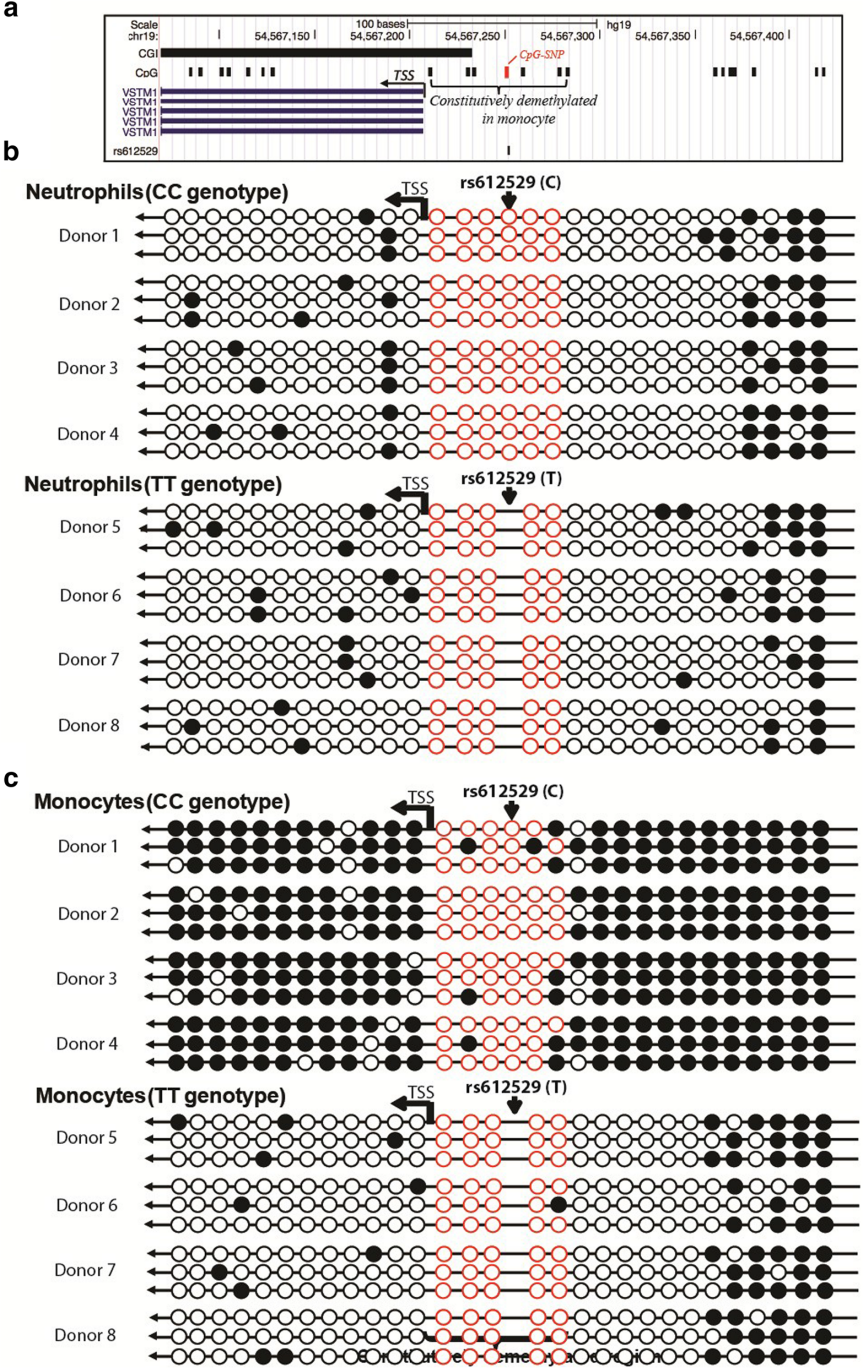
Allele and cell type-dependent methylation of the *VSTM1* gene promoter. **a** *Schematic overview* of the analyzed 693 bp region of the VSTM1 gene locus (–1 to –388 upstream and +1 to +305 downstream to TSS). The location of a predicted CGI is indicated together with the position of individual CpG pairs (*solid black lines*); the CpG-SNP formed by rs612526 is indicated by a *red box*. The location of the first exon in the transcript variants of *VSTM1* (covered in the C-methylation assay) is represented by *solid blue vertical lines* (the sequence of the 693 bp region is shown in Additional file 3). **b** Allele-independent C-methylation in neutrophils. The methylation of CpG pairs in the 693 bp region was analyzed by bisulfite sequencing. The methylation state of each CpG element is represented by *circles* (*full circle*: methylated, *empty circle*: demethylated); the location of the TSS as well as of rs612529 is indicated. The methylation state in the cells is shown as a set of three independently sequenced clones each obtained from four different donors of the rs612529 CC genotype (donors 1–4) and the TT genotype (donors 5–8). *Red circles* indicate CpG pairs of the constitutively demethylated core region. **c** Allele-dependent methylation in monocytes. The CpG-methylation state is shown for monocytes, which were isolated from the same donors described above for the neutrophils

Epigenetic mechanisms facilitate the response to the changes in the environment through modulating the gene expression. For example, the methylation of CpG pairs triggers condensation of the DNA, which affects the interaction with transcription factors and other regulatory factors controlling gene expression [26]. This mechanism seems to be relevant also for *VSTM1* as in lymphoma cells (which do not exhibit any SIRL-1 expression) the silencing of the gene is associated with the methylation of some CpG pairs located within the first exon [27]. An in silico scan (http://rafalab.jhsph.edu/CGI/) [25] suggested the presence of a CGI at the start of *VSTM1*. It completely covered exon 1, extending from the promoter region towards intron 1 (Fig. 5a). A segment of that region of about 700 bp (411 bp upstream and 282 bp downstream of the TSS contained a total of 30 CpG pairs including one CpG-SNP formed by the C allele of rs612529 (Additional file 3).

In order to study a potential role of the rs612529 CpG-SNP in the control of the monocyte-specific SIRL-1 expression, we analyzed cytosine methylation by bisulfite sequencing. A total of eight donors (four CC genotype and four TT genotype) were used to determine the influence of the allelic state in both eQTL relevant monocytes and allele-independent neutrophils (Fig. 5b and c). While the CpG-SNP itself would appear to be a potential site for an allele-specific methylation, it was apparently not a target for modification, as it was found to be located in a 54-bp core region containing six additional CpG pairs, which were constitutively demethylated in all samples analyzed. While the CpG-SNP was not directly affected by epigenetic modifications, it had a strong and cell-type–specific effect on the general methylation state in the analyzed gene region. In line with the bright but allele-independent surface expression detected by FACS (compare Fig. 2) neutrophils exhibit demethylated CpG elements both in the CC and the TT donors (Fig. 5b). In monocytes, however, only TT donors had a demethylated *VSTM1* promoter, while the gene segment was almost completely methylated in CC donors (Fig. 5c). Thus, in monocytes the formation of a CpG pair by the C allele of rs612529 seems to promote the methylation of other CpG pairs in the region results in a silencing of the locus (compare Fig. 2).

#### Genetic association of rs612529 to AD phenotype

From earlier reports, it was hypothesized that allergic and inflammatory skin diseases are linked to oxidative stress [28, 29]. Since SIRL-1 acts as a balancing factor for the antibody-induced ROS production by monocytes, we evaluated whether the allelic state of rs612529 had any influence on the manifestation of inflammatory skin diseases. We used an existing allergy cohort for rs612529 genotype association studies [20, 30] (Additional file 1). Association analysis for rs612529 in 324 clinically diagnosed AD cases and 486 healthy controls identified a significant association of *P* < 0.05 with the C allele present at a higher frequency in patients than in healthy controls (OR = 1.3; *P* = 4.00 × 10^–6^). The same trend was also observed in two larger cohorts, one Chinese [21] and one Caucasian [22] (Table 1). Upon combining all three independent cohorts, the association of rs612529 to AD risk remained significant with a Meta *P* value of 1.14 × 10^–6^ in a combined sample size of 2536 AD cases against 4118 controls. Thus, the low surface expression of SIRL-1 in monocytes associated with the C allele potentially increases risk for inflammatory skin diseases such as dermatitis.

**Table 1:** Association of rs612529 with AD

References^a^	Population^b^	Cases^c^ (n)	Controls^d^ (n)	Effect allele^e^	Effect allele frequency^f^	OR (95% CI)^g^	*P* value^h^
[15]	Caucasian	1200	2270	C	0.209	1.12 (1.15–1.29)	1.26 × 10^–3^
[14]	Chinese	1012	1362	C	0.322	1.15 (1.07–1.30)	2.6 × 10^–2^
[12, 13]	Chinese	324	486	C	0.36	1.29 (1.10–1.45)	4 × 10^–6^
	Combined	2536	4118				**1.14 × 10** ^**–6**^

^a^Initial study describing the cohorts used for association

^b^Ethnicity of the population under study

^c^Number of atopic dermatitis cases

^d^Number of controls

^e^Allele used for the calculating the odds ratio statistics

^f^Allele frequency of the effect allele in the individual study population

^g^Odds ratio estimated with 95% confidence interval

^h^ *P* value estimated for the case control association study for atopic dermatitis phenotype in the individual study population

Meta *P* value is the *P* value calculated for all three cohorts combined using the Stoufier’s z trend meta-analysis which considers individual study *P* value sample sizes and effect directions using Meta *P* program [20]

## Discussion

In this study, we significantly associate the previously reported cell-type–specific variations in SIRL-1 expression to a genetic cause. A single SNP located in the promoter region mediates the epigenetic silencing of the entire gene locus, a process presumably mediated by the allele-specific recruitment of PU.1/YY1. The allelic effect is particularly evident in monocytes, where the C allele of rs612529 is associated with a near complete lack of SIRL-1 expression. This in turn results in a diminished control of pro-inflammatory reactions, evidenced by the failure of SIRL-1 agonists to dampen the production of Fc-receptor induced ROS in these cells. Genetic association results in independent cohorts suggest that this is associated consistently with an increased risk of AD.

We then demonstrated allele-specific binding of PU.1 to the promoter region of *VSTM1* using follow-up functional experiments such EMSA and ChIP assays. This transcription factor PU.1 has been shown to be a master regulator of myeloid cell development, which could explain the observed specificity of the SIRL-1 expression in cells of this lineage [31–33]. PU.1 forms complexes with various regulatory factors chromatin remodelers, including DNA methyltransferases and demethylases to activate or repress transcription [34]. A recent study has shown that in non-differentiated monocytes, PU.1 can act as docking site for the demethylase, Tet2 [35]. In these cells the recruitment of Tet2 by PU.1 leads to the targeted demethylation of the respective gene locus. In line with this observation we therefore hypothesize that the allele-dependent methylation of the *VSTM1* gene reported here can be explained by the conditional recruitment of Tet2 or other demethylases as a result of the allele-specific binding of PU.1 to rs612529 (Additional file 8). In monocytes rs612529 would thus represent a genetic master switch for the epigenetic control of SIRL-1 expression by CpG demethylation.

Mechanistically, the methylation of CpG promotes the compaction of chromatin by providing interaction sites for methyl-CpG-binding proteins (MBPs) [24, 36, 37]. Hence, CpG-SNPs, which allow methylation in an allele-dependent way, are often associated with function [38–40]. In this context, it is interesting to note that rs612529 is a CpG-SNP where the C allele forming the CpG pair is associated with the epigenetic silencing of the *VSTM1* gene. However, in contrast to conventional CpG-SNPs, it is not a direct target for methylation. Instead of acting as a simple “switch” to close the chromatin, it rather functions as a genetic/epigenetic regulator that modulates the DNA methylation state by the recruitment of CpG modifying enzymes. In this way, the effect of the CpG-SNP becomes potentiated, as the methylation affects not just a single CpG but also many other CpG sites in its vicinity. Subtle changes associated with the allelic switching between C and T therefore translate into extensive epigenetic alterations of the gene, which makes rs612529 a master genetic regulator of the *VSTM1* gene in monocytes.

We then evaluated if the causal SNP we identified, rs612529, has potential pathological implications, given the striking variations in SIRL-1 surface expression observed on monocytes. By using the allelic states of the SNP as proxy for the expression levels in these cells we could establish AD as being associated with a low SIRL-1 expression in these cells. AD is a chronic inflammatory skin disease affecting 60 million people in Europe and the USA [41]. The pathophysiology of AD is a result from a complex interplay of host genetic factors, environmental triggers, skin integrity, immune balance, and the skin microbiome [42]. Thus, a tight control of the immune response is needed to ensure effective pathogen defense without collateral damage to the protective skin layers. IgE is one of the determinants predisposing factor for AD. However, earlier reports have shown that in early childhood, elevated levels of IgE do not seem to correlate with disease manifestations or severity of AD [43]. Moreover, some evidence pointing towards a possible role of IgA derives from an earlier report indicating that patients with selective IgA deficiency have an increased incidence of atopic dermatitis [44].

SIRL-1 is an effective inhibitor of monocyte-derived pro-inflammatory reactions such as FcR-induced ROS production. In our study, we show that blood monocytes represent a heterogeneous population characterized by different Fc receptors. In Fig. 3a and Additional file 5, FcαR+ monocytes co-segregate with a high expression of SIRl-1 expression. This is not observed in FcγRIII+ and FcεR+ monocytes, the latter being expressed on a very small subset of monocytes. As such, we examined if co-activating of FcαR and SIRL-1 with human IgA and anti-SIRL-1 mAb would influence the capacity of monocytes to generate ROS. We show the capacity of SIRL-1 to modulate ROS production in monocytes is a function of VSTM1 genotype. The association of the low expressing C allele with AD thus leads us to hypothesize that inhibitory regulation by SIRL-1 of monocytes or monocyte-derived cells plays a key role in skin homeostasis.

## Conclusion

In summary, our study introduces with rs612529 an important genetic factor influencing the risk for skin inflammation. The identification of this link was made possible by defining an eQTL associated with these fluctuations from the whole blood data of our Chinese cohort and using the genotypes to mine pre-existing genetic association databases. A growing number of eQTL and GWAS data have been published already but only a fraction of the GWAS datasets are publicly accessible so far. Given the power the approach has to uncover new and unexpected links between genetically controlled phenotypes and disease manifestation, it appears to be mandatory that more of the “large data,” including eQTL and GWAS, has to be compiled and curated in easily accessible repositories which are open for the general scientific community. In the case of SIRL-1 it not only helped to understand the role of the receptor in controlling immune cells, it also identified a novel candidate target for the treatment of AD. The identification of the as-yet unknown ligand(s) of SIRL-1 may thus open up new avenues to control or suppress the manifestation of the inflammatory skin disease.

## Additional files

Additional file 1: Cohorts used in the study. The data for this study were collected from seven different cohorts. Four of these cohorts were established for the functional association studies (eQTL, FACS analysis, ROS assay, DNA methylation, and ChiP assay), while the three other cohorts were used for the genetic association studies with atopic dermatitis (AD). Size of the cohorts as well as ethnicity and appropriate reference are provided for each cohort. (PPTX 80 kb)
Additional file 2: Oligonucleotide sequences of probes used in EMSA experiments. The table lists the sequence of oligonucleotides used as radiolabeled probes or as cold competitors in EMSA. VSTM1 C and T probe sequences represent the two allelic variants of a 25-bp region in the VSTM1 promoter containing the rs612529 SNP. Consensus and mutant oligonucleotides are based on consensus binding sites of the respective transcription factor. (PDF 51 kb)
Additional file 3: VSTM1 promoter sequence. The sequence of the region 411 bases upstream of the TSS and 282 bases downstream (untranslated region, Exon1, and part of Intron 1 of VSTM1 transcript) was obtained from UCSC genome browser (https://genome.ucsc.edu/). The location of rs612529 T/C (underlined) as well as of each CpG site is indicted (*red bold font*). The transcription start site is indicated by “TSS,” the translational start by the start codon ATG. The transcribed region is indicated by small letters. Primers for bisulfite sequencing (F1/F2 and B1/B2) as well as the primer pair used for rs612529 genotyping and ChiP analysis are indicated by empty arrows. (PDF 161 kb)
Additional file 4: Gating strategy for the flow cytometry analysis of SIRL-1 expression on whole blood samples. Whole blood samples stained with antibodies specific for CCR3, CD16, CD123, CD14, FcεRIα, and HLA-DR were counterstained with either anti-SIRL-1 or isotype-matched control antibody. Gates allowing the simultaneous analysis of the SIRL-1 staining on neutrophils, eosinophils, basophils, monocytes, mDC, pDC, B cells, and NK & T cells are indicated. (PDF 210 kb)
Additional file 5: Gating strategy for monocytes expressing FcγRIII, FcεRIα, or FcαR. PBMCs were stained with antibodies specific for FcγRIII (CD16), FceRIα, FcαR (CD89), and CD14 together with anti-SIRL-1, or isotype-matched control antibody. Monocytes were gated based on scatter properties (SSC-A and FSC-A) and live/dead-staining (AmCyan). Gates for FcγRIII+ monocytes, FcεRIα + monocytes, and FcαR+ monocytes are indicated in plots displaying the CD14 staining vs. the respective Fc-receptor. (PDF 297 kb)
Additional file 6: In silico prediction of transcription factors with allele-specific binding to rs612529. The web based prediction tools JASPAR (**A**) and TRANSFAC (**B**) were used to identify potential transcription factor binding sites in the VSTM1 promoter region affected by the allelic variations of rs612529 T/C. Only sites predicted with a binding score greater than 5.0 (JASPAR) and 0.9 (TRANSFAC) were selected for further analysis. The nucleotide underlined in *red* indicates the location of rs612529 within binding motif of the respective TF. *Yellow shaded rows* indicate transcription factors predicted to be highly specific for T allele.5. (PDF 55 kb)
Additional file 7: EMSA competition of PU.1 (ETS family) and YY1 consensus probes with rs612529 probes. EMSA experiments were performed with radiolabeled probes representing the consensus binding sequence of PU.1 and YY1. **A** EMSA competition experiment with consensus PU.1-probe. Nuclear extracts from primary monocytes were exposed to radiolabeled PU.1 consensus alone (lane 1) or together with excess of unlabeled PU.1 consensus probes (lane 2), PU.1 mutant probes (lane 3), or increasing amounts of rs612529 C (lanes 4–6) or T probes (lanes 7–9). The competitors were added to the binding reactions at increasing concentrations prior to the incubation with probe. **B** EMSA competition experiment with consensus YY1-probe. Radiolabeled YY1 probe was used alone (lane 1) or with excess of unlabeled YY1 probe (lane 2), YY1 mutant probe (lane 3), or increasing amounts of rs612529 C (lanes 4–7) or T probes (lanes 8–11). (PDF 243 kb)
Additional file 8: Model on the potentiating effect of a regulatory CpG-SNP. **A** Conventional vs. regulatory CpG-SNPs. The methylation of CpG pairs results in the condensation of chromatin due to a tighter packaging of the histone-DNA complexes (*open circle*: non-methylated CpG, *closed circle*: methylated CpG). While a conventional CpG-SNP can assist this process by providing only one additional allele-dependent methylation site (*middle panel*), a regulatory CpG-SNP (such as rs612529) modulates the recruiting of CpG-modifying enzymes (*right panel*). In the case of rs612529, this seems to be mediated by the allele-specific binding of PU.1 to the T allele of the CpG-SNP. In monocytes PU.1 was reported to bind the demethylase Tet-2. **B** Potentiating effect. Recruitment of Tet-2 mediates the chromatin-opening by facilitating the demethylation of all CpG pairs in the vicinity. The epigenetic effect is allele-specific (T allele) and direction of the direction probably cell-type–dependent. (PDF 415 kb)
