# Supplementary material for: A functional SNP associated with atopic dermatitis controls cell type-specific methylation of the VSTM1 gene locus
Source: Genome Med. 2017 Feb 20;9:18. doi: 10.1186/s13073-017-0404-6 (PMC5319034; doi:10.1186/s13073-017-0404-6)
Supplement: Additional file 1: — Cohorts used in the study. The data for this study were collected from seven different cohorts. Four of these cohorts were established for the functional association studies (eQTL, FACS analysis, ROS assay, DNA methylation, and ChiP assay), while the three other cohorts were used for the genetic association studies with atopic dermatitis (AD). Size of the cohorts as well as ethnicity and appropriate reference are provided for each cohort. (PPTX 80 kb) [file 13073_2017_404_MOESM1_ESM.pptx]

## Slide 1
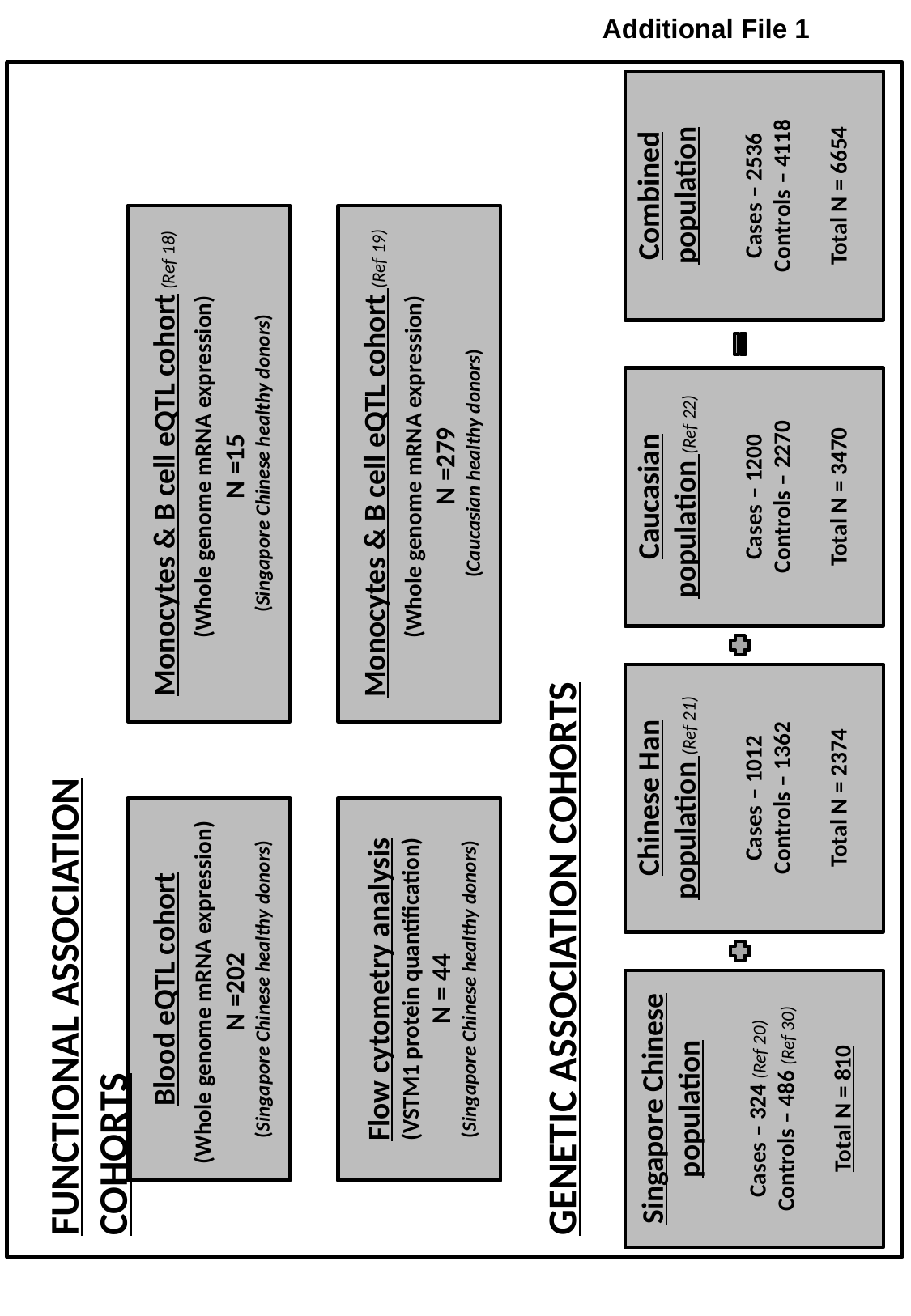

Additional File 1
Functional Association cohorts
Blood eQTL cohort
(Whole genome mRNA expression)
N =202
(Singapore Chinese healthy donors)
Monocytes & B cell eQTL cohort (Ref 18)
(Whole genome mRNA expression)
N =15
(Singapore Chinese healthy donors)
Flow cytometry analysis
(VSTM1 protein quantification)
N = 44
(Singapore Chinese healthy donors)
Monocytes & B cell eQTL cohort (Ref 19)
(Whole genome mRNA expression)
N =279
(Caucasian healthy donors)
Genetic Association cohorts
Singapore Chinese population
Cases – 324 (Ref 20)
Controls – 486 (Ref 30)
Total N = 810
Chinese Han population (Ref 21)
Cases – 1012
Controls – 1362
Total N = 2374
Caucasian population (Ref 22)
Cases – 1200
Controls – 2270
Total N = 3470
Combined
population
Cases – 2536
Controls – 4118
Total N = 6654
