# Supplementary material for: A functional SNP associated with atopic dermatitis controls cell type-specific methylation of the VSTM1 gene locus
Source: Genome Med. 2017 Feb 20;9:18. doi: 10.1186/s13073-017-0404-6 (PMC5319034; doi:10.1186/s13073-017-0404-6)
Supplement: Additional file 2: — Oligonucleotide sequences of probes used in EMSA experiments. The table lists the sequence of oligonucleotides used as radiolabeled probes or as cold competitors in EMSA. VSTM1 C and T probe sequences represent the two allelic variants of a 25-bp region in the VSTM1 promoter containing the rs612529 SNP. Consensus and mutant oligonucleotides are based on consensus binding sites of the respective transcription factor. (PDF 51 kb) [file 13073_2017_404_MOESM2_ESM.pdf]

Additional file 2

(a)	Oligonucleotides for EMSA probe and competition experiments	
EMSA Probes and competitors	Sequence 5' to 3' direction	Design of EMSA Probes and competitors based on
VSTM1_C Probe	Forward – 5'-ACACCGGATTCA***C*** GGGGAAGAAGTT-3'	C allele of rs612529
	Reverse – 5'-AACTTCTTCCCC***G*** TGAATCCGGTGT-3	
VSTM1_ T Probe	Forward – 5'-ACACCGGATTCA***T*** GGGGAAGAAGTT-3'	T allele of rs612529
	Reverse – 5'-AACTTCTTCCCC***A*** TGAATCCGGTGT-3'	
YY1_Consensus	Forward – 5'-CGCTCCCCG***GCC*** ATCTTGGCGGCTGGT-3'	Santa Cruz Biotech. (Cat. Nr. - SC-2533)
	Reverse – 5'-ACCAGCCGCCAAGAT***GGC*** CGGGGAGCG-3'	
YY1_Mutant	Forward – 5'-CGCTCCCCGATTATCTTGGCGGCTGGT-3'	Santa Cruz Biotech. (Cat. Nr. - SC-2534)
	Reverse – 5'-ACCAGCCGCCAAGATAATCGGGGAGCG-3'	
MZF1_Consensus 1	Forward – 5'-GATCTAAAA***GTGGGG*** AGAAAA-3'	TRANSFAC predition
	Reverse – 5'-TTTTCT***CCCCAC*** TTTTAGATC-3'	
MZF1_Mutant 1	Forward – 5'-GATCTAAAA***GTTTTT*** AGAAAA-3'	TRANSFAC predition
	Reverse – 5'-TTTTCT***AAAAAC***TTTTAGATC-3'	
MZF1_Consensus 2	Forward – 5'- GATCCGGCTGGTGA***GGGGG*** AATCG-3'	TRANSFAC predition
	Reverse – 5'- CGATT***CCCCC*** TCACCAGCCGGATC-3'	
MZF1_Mutant 2	Forward – 5'- GATCCGGCTGGTGA***TTTTT*** AATCG-3'	TRANSFAC predition
	Reverse – 5'- CGATT***AAAAA*** TCACCAGCCGGATC-3'	
PU.1 consensus (Ets family_Consensus)	Forward – 5'-GGGCTGCTTGAG***GAA*** GTATAAGAAT	Santa Cruz Biotech. (Cat. Nr. - SC-2549)
	Reverse – 5'-ATTCTTATAC***TTC*** CTCAAGCAGCCC	
PU.1 consensus (Ets family_Mutant)	Forward – 5'-GGGCTGCTTG***AGA*** GAGTATAAGAAT	Santa Cruz Biotech. (Cat. Nr. -SC-2550)
	Reverse – 5'ATTCTTATAC***TCT*** CTCAAGCAGCCC	
PBX1_Consensus	Forward – 5'-CTCCAATTAGTGCATCAA***TC*** AATTCG	Santa Cruz Biotech. (Cat. Nr. - SC-2581)
	Reverse – 5'-CGAATT***GA*** TTGATGCACTAATTGGAG	
PBX1_Mutant	Forward – 5'-CTCCAATTAGTGCATCAA***GG*** AATTCG	Santa Cruz Biotech. (Cat. Nr. - SC-2582)
	Reverse – 5'-CGAATT***CC*** TTGATGCACTAATTGGAG	
