# Supplementary figures and images for: A functional SNP associated with atopic dermatitis controls cell type-specific methylation of the VSTM1 gene locus
Source: Genome Med. 2017 Feb 20;9:18. doi: 10.1186/s13073-017-0404-6 (PMC5319034; doi:10.1186/s13073-017-0404-6)

# VSTM1 Promoter sequence with primers and CpG information

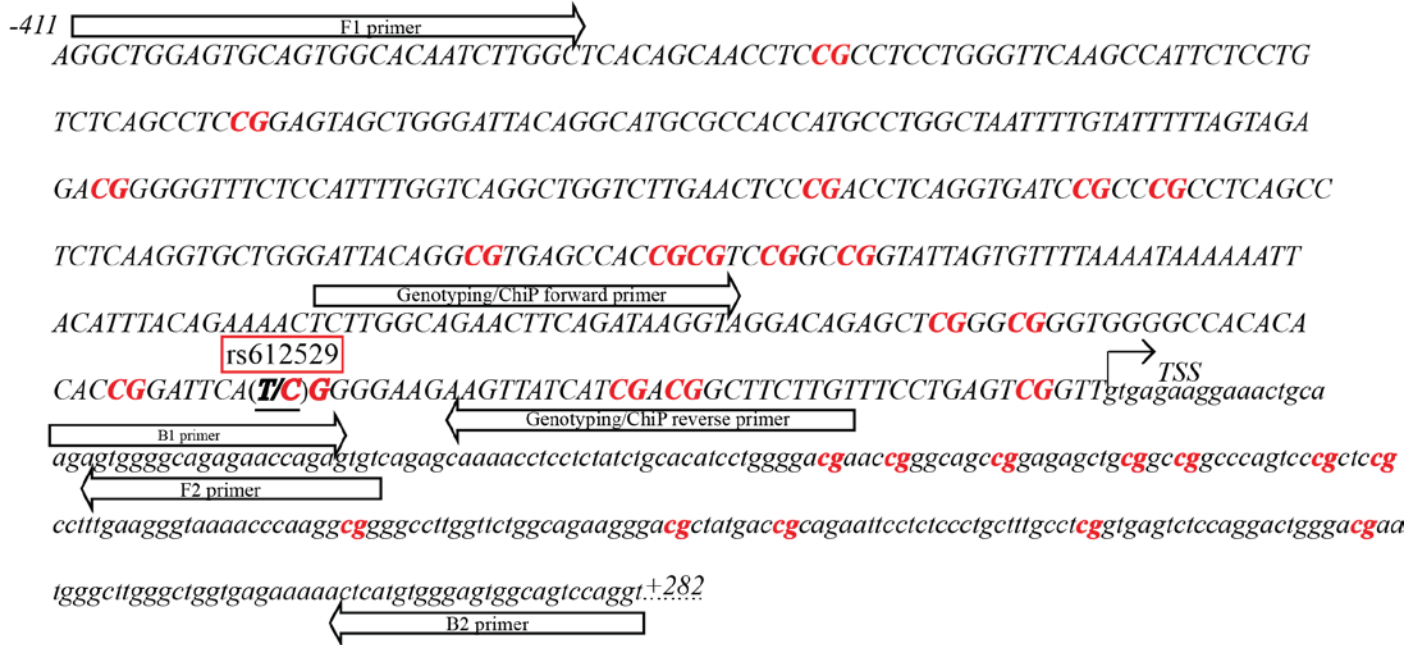

Supplement: Additional file 3: — VSTM1 promoter sequence. The sequence of the region 411 bases upstream of the TSS and 282 bases downstream (untranslated region, Exon1, and part of Intron 1 of VSTM1 transcript) was obtained from UCSC genome browser (https://genome.ucsc.edu/). The location of rs612529 T/C (underlined) as well as of each CpG site is indicted (red bold font). The transcription start site is indicated by “TSS,” the translational start by the start codon ATG. The transcribed region is indicated by small letters. Primers for bisulfite sequencing (F1/F2 and B1/B2) as well as the primer pair used for rs612529 genotyping and ChiP analysis are indicated by empty arrows. (PDF 161 kb) [file 13073_2017_404_MOESM3_ESM.pdf]

## Additional file 4

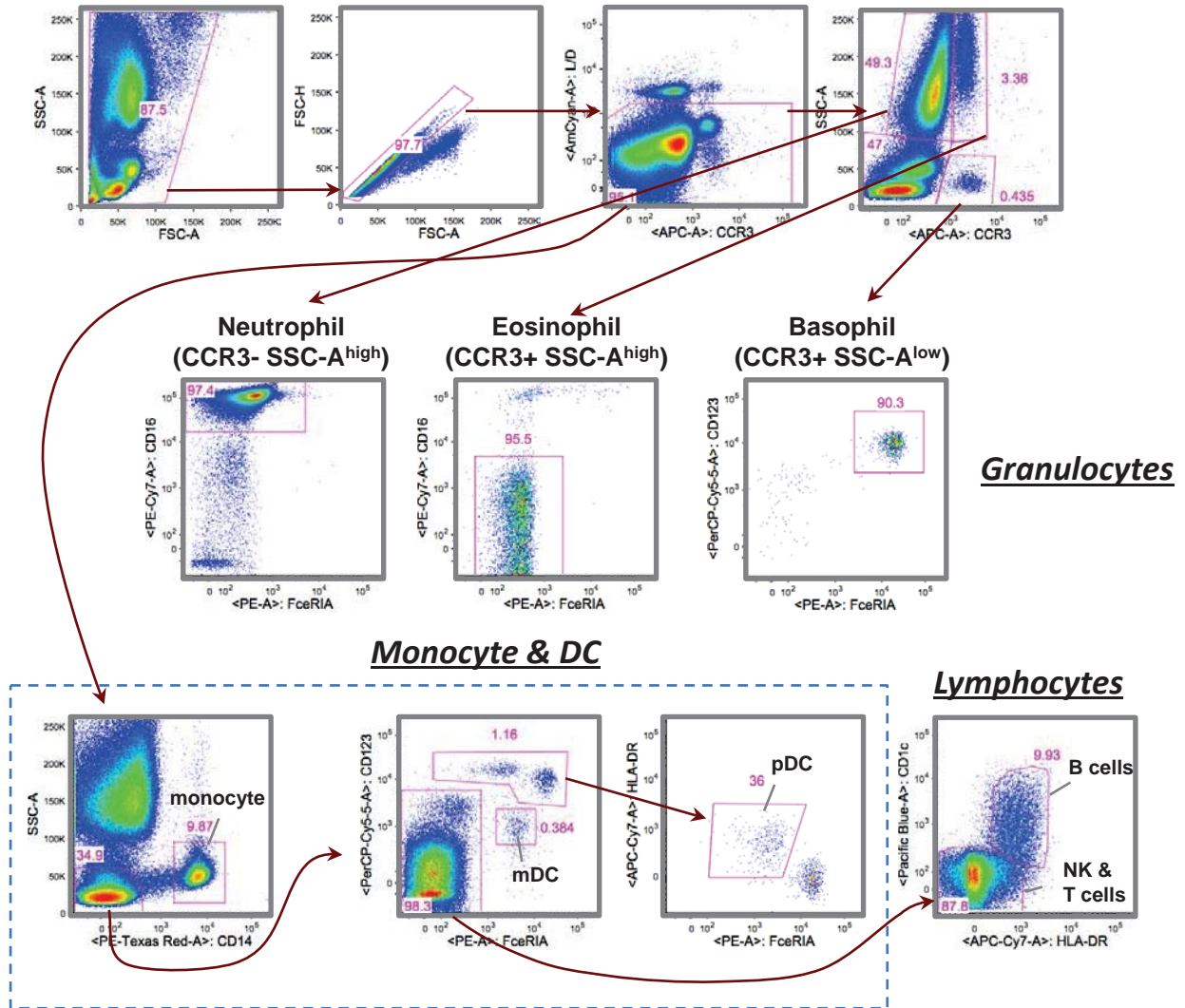

Supplement: Additional file 4: — Gating strategy for the flow cytometry analysis of SIRL-1 expression on whole blood samples. Whole blood samples stained with antibodies specific for CCR3, CD16, CD123, CD14, FcεRIα, and HLA-DR were counterstained with either anti-SIRL-1 or isotype-matched control antibody. Gates allowing the simultaneous analysis of the SIRL-1 staining on neutrophils, eosinophils, basophils, monocytes, mDC, pDC, B cells, and NK & T cells are indicated. (PDF 210 kb) [file 13073_2017_404_MOESM4_ESM.pdf]

## Additional file 5

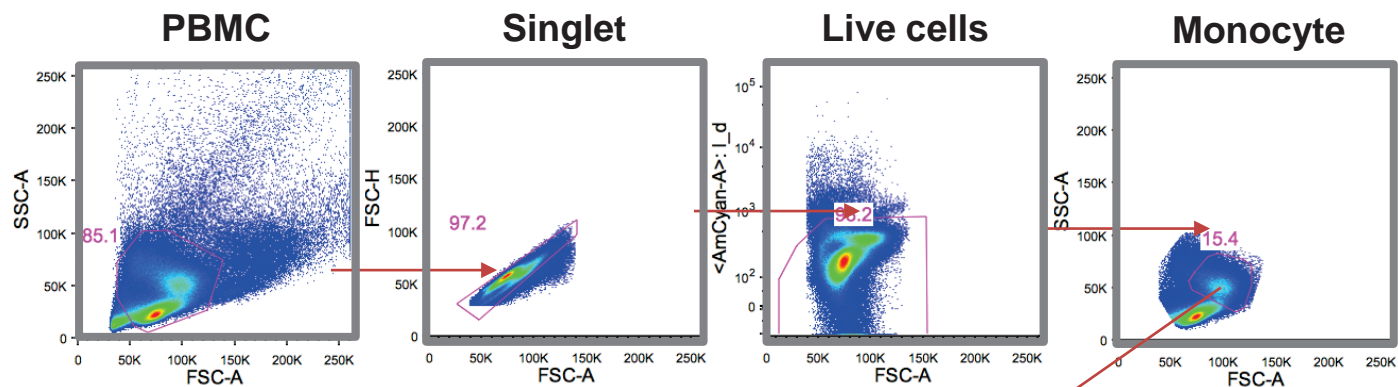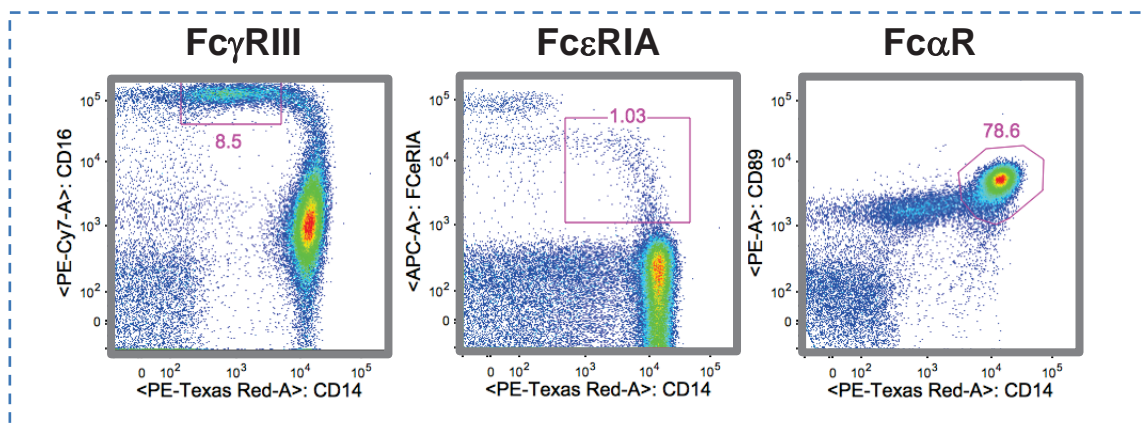

Supplement: Additional file 5: — Gating strategy for monocytes expressing FcγRIII, FcεRIα, or FcαR. PBMCs were stained with antibodies specific for FcγRIII (CD16), FceRIα, FcαR (CD89), and CD14 together with anti-SIRL-1, or isotype-matched control antibody. Monocytes were gated based on scatter properties (SSC-A and FSC-A) and live/dead-staining (AmCyan). Gates for FcγRIII+ monocytes, FcεRIα + monocytes, and FcαR+ monocytes are indicated in plots displaying the CD14 staining vs. the respective Fc-receptor. (PDF 297 kb) [file 13073_2017_404_MOESM5_ESM.pdf]

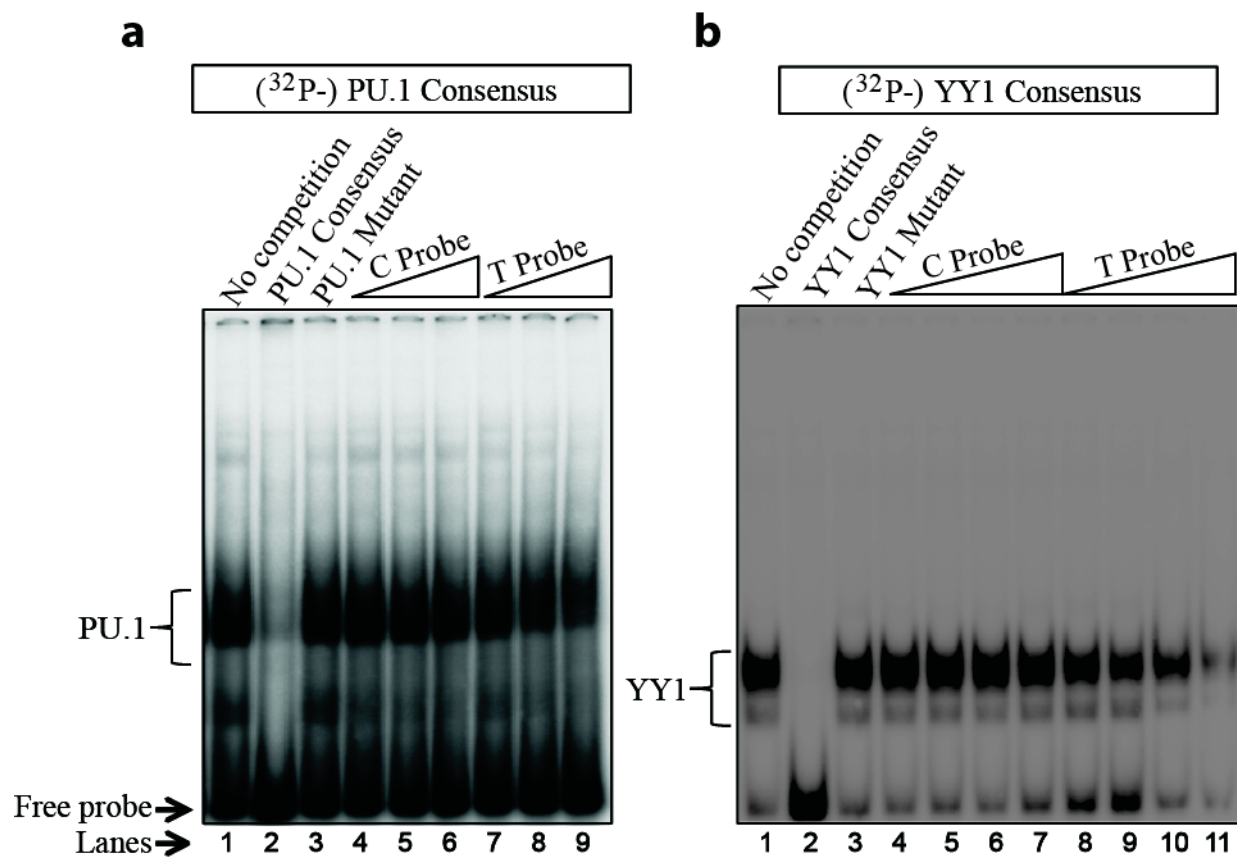

Supplement: Additional file 7: — EMSA competition of PU.1 (ETS family) and YY1 consensus probes with rs612529 probes. EMSA experiments were performed with radiolabeled probes representing the consensus binding sequence of PU.1 and YY1. A EMSA competition experiment with consensus PU.1-probe. Nuclear extracts from primary monocytes were exposed to radiolabeled PU.1 consensus alone (lane 1) or together with excess of unlabeled PU.1 consensus probes (lane 2), PU.1 mutant probes (lane 3), or increasing amounts of rs612529 C (lanes 4–6) or T probes (lanes 7–9). The competitors were added to the binding reactions at increasing concentrations prior to the incubation with probe. B EMSA competition experiment with consensus YY1-probe. Radiolabeled YY1 probe was used alone (lane 1) or with excess of unlabeled YY1 probe (lane 2), YY1 mutant probe (lane 3), or increasing amounts of rs612529 C (lanes 4–7) or T probes (lanes 8–11). (PDF 243 kb) [file 13073_2017_404_MOESM7_ESM.pdf]

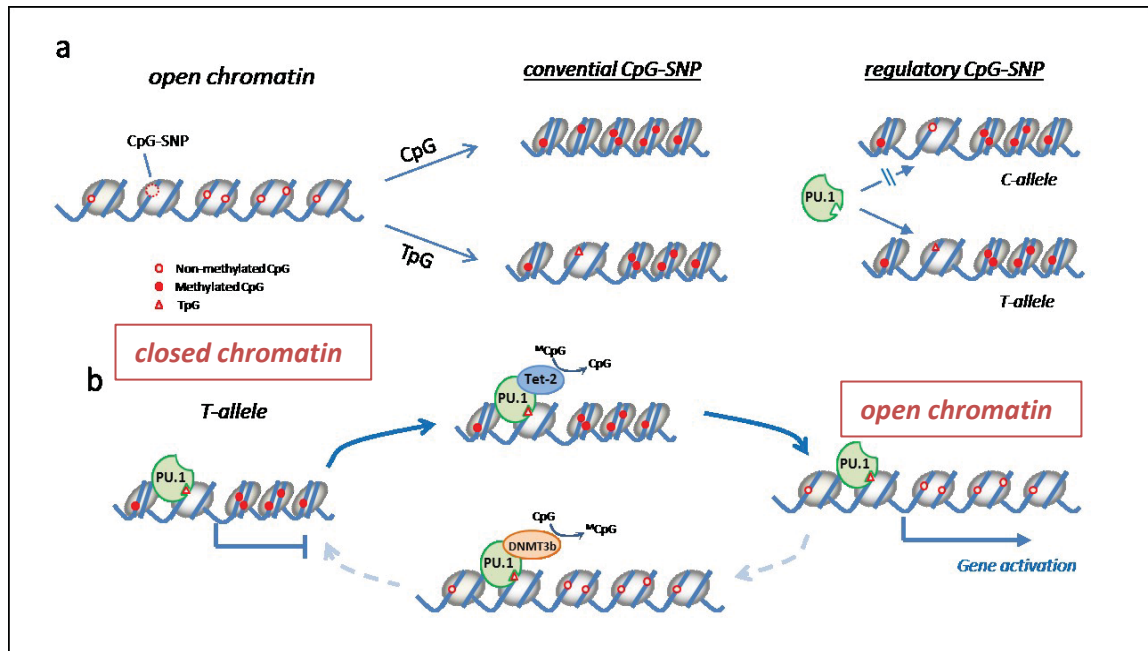

Supplement: Additional file 8: — Model on the potentiating effect of a regulatory CpG-SNP. A Conventional vs. regulatory CpG-SNPs. The methylation of CpG pairs results in the condensation of chromatin due to a tighter packaging of the histone-DNA complexes (open circle: non-methylated CpG, closed circle: methylated CpG). While a conventional CpG-SNP can assist this process by providing only one additional allele-dependent methylation site (middle panel), a regulatory CpG-SNP (such as rs612529) modulates the recruiting of CpG-modifying enzymes (right panel). In the case of rs612529, this seems to be mediated by the allele-specific binding of PU.1 to the T allele of the CpG-SNP. In monocytes PU.1 was reported to bind the demethylase Tet-2. B Potentiating effect. Recruitment of Tet-2 mediates the chromatin-opening by facilitating the demethylation of all CpG pairs in the vicinity. The epigenetic effect is allele-specific (T allele) and direction of the direction probably cell-type–dependent. (PDF 415 kb) [file 13073_2017_404_MOESM8_ESM.pdf]
