# Supplementary material for: A functional SNP associated with atopic dermatitis controls cell type-specific methylation of the VSTM1 gene locus
Source: Genome Med. 2017 Feb 20;9:18. doi: 10.1186/s13073-017-0404-6 (PMC5319034; doi:10.1186/s13073-017-0404-6)
Supplement: Additional file 6: — In silico prediction of transcription factors with allele-specific binding to rs612529. The web based prediction tools JASPAR (A) and TRANSFAC (B) were used to identify potential transcription factor binding sites in the VSTM1 promoter region affected by the allelic variations of rs612529 T/C. Only sites predicted with a binding score greater than 5.0 (JASPAR) and 0.9 (TRANSFAC) were selected for further analysis. The nucleotide underlined in red indicates the location of rs612529 within binding motif of the respective TF. Yellow shaded rows indicate transcription factors predicted to be highly specific for T allele.5. (PDF 55 kb) [file 13073_2017_404_MOESM6_ESM.pdf]

Additional file 6

a) JASPAR (score > 5.0)

predicted TF	probe	site	score	probe	site	score
SOX10	T	CGGTGT	5.2	C	CGGTGT	5.2
SPIB	T	ACCGGAT	5.2	C	ACCGGAT	5.2
Hoxc9	T	CCCCATGAATCCG	7.7	C	-	-
Hoxa9	T	CCCATGAATCC	10.3	C	CCCGTGAATCC	6.4
JUN::FOS	T	TGAATCC	5.5	C	TGAATCC	5.5
Pax2	T	ATTCATGG	5.7	C	ATTCACGG	6.3
Arnt	T	-	-	C	CCCGTG	6.1
Spi1 (**PU.1**)	T	TTCATGGGGAAGAA	5.3	C	-	-
MZF1 (5-13)	T	TCATGGGGAA	7.3	C	-	-
Mycn	T	-	-	C	TCCCCGTG	5
MZF1 (1-4)	T	TGGGGA	9	C	CGGGGA	8.5
SPIB	T	TGGGGAA	8.1	C	CGGGGAA	4.6
ELF5	T	TTCTTCCCC	5.5	C	TTCTTCCCC	5.5
NFATC2	T	TCTTCCC	6.4	C	TCTTCCC	6.4
EHF	T	ACTTCTTC	6.5	C	ACTTCTTC	6.5

b) TRANSFAC (score > 0.9)

predicted TF	probe	site	score	probe	site	score
V$CETS1P54_02	T	acacCGGATtcat	0.951	C	acacCGGATtcac	0.95
V$CETS1_01	T	aCCGGAttca	0.922	C	aCCGGAttca	0.92
V$CETS1P54_01	T	aCCGGAttca	0.917	C	aCCGGAttca	0.92
V$CETS2_01	T	acCGGATtca	0.908	C	acCGGATtca	0.91
V$CMAF_02	T	ATTCA	0.957	C	ATTCA	0.96
V$CEBP_Q3	T	ttcatgGGGAAg	0.955	C	ttcacgGGGAAg	0.93
V$YY1_Q6_03	T	ttCATGG	0.946	C	-	-
V$SPI1_01	T	ttcatggGGAAGaagtt	0.93	C	ttcacggGGAAGaagtt	0.92
V$MZF1_01	T	catGGGGA	0.983	C	cacGGGGA	0.98
V$IK2_01	T	catGGGGAagaa	0.948	C	cacGGGGAagaa	0.94
V$CETS1P54_02	T	catgGGGAAgaag	0.936	C	cacgGGGAAgaag	0.9310
V$IK1_01	T	catgGGGAAgaag	0.905	C	cacgGGGAAgaag	0.90
V$MZF1_Q5	T	tGGGGAa	0.993	C	-	-
V$SPI1_03	T	tggGGAAGaa	0.959	C	cggGGAAGaa	0.96
V$PU1_Q6	T	tggGGAAG	0.952	C	cgGGGAAgaa	0.94
V$SPI1_02	T	tgGGGAAgaa	0.941	C	cggGGAAG	0.93
V$ETS2_Q6	T	ggGGAAG	0.948	C	ggGGAAG	0.95
V$ETS_Q6	T	ggGGAAGa	0.938	C	ggGGAAGa	0.94
V$CETS1_Q6	T	gGGAAGa	0.932	C	gGGAAGa	0.93
V$SPI1_Q5	T	GGGAAg	0.922	C	GGGAAg	0.92
V$HMGIY_Q4	T	gAAGAA	0.998	C	gAAGAA	1.00
